# Orthopædia

**Published:** 1875-06

**Authors:** 


*Orthopœdia; or a Practical Treatise on the Aberrations of the Human Form.* By James Knight, M. D. New York: G. P. Putnam's Sons.

Dr. Knight has presented this work to the profession as the result of his experience with a large class of cases of which it treats. As Surgeon to the New York Hospital for the Ruptured and Crippled, he has naturally had a large amount of practice in orthopædic surgery. The work is divided into twelve chapters, which consider in turn, defective physical formation, impairment of tissues resulting in contortions, general remarks upon the treatment of talipes, infantile paralysis, electricity as a therapeutic agent in paralysis, contractions of the hands, fingers and toes, lateral curvature of the spine, torticollis, rachitis, hernia, procedentia uteri, ectropium vesicæ, relaxed abdomen, varicose veins, bursæ ganglion, pathological consideration of diseases of joints, diseases of bones, and tonics and their effects upon the system. It is rather difficult to reconcile the appearance of some of the topics named in a work upon orthopædia, but as the author has given them in the main a very intelligent consideration, we will not question the cause of their appearance.

The illustrations are in general good, though some seem to be roughly made. We have read with some care portions of the work upon the general treatment of deformities, and find that we coincide with what the author says in most instances. The author does not dwell with the force that he might upon the influence of uterine pressnre, and deficient supply of amniotic fluid in the production of talipes. We have seen several cases where there seemed to be conclusive evidence that this was the sole cause of the deformity. To surgeons interested in this class of cases, the work will possess considerable interest.

